# Effectiveness of Herbal Essential Oils as Single and Combined Repellents against *Aedes aegypti*, *Anopheles dirus* and *Culex quinquefasciatus* (Diptera: Culicidae)

**DOI:** 10.3390/insects13070658

**Published:** 2022-07-21

**Authors:** Nataya Sutthanont, Monthatip Sudsawang, Theerawit Phanpoowong, Patchara Sriwichai, Jiraporn Ruangsittichai, Chawarat Rotejanaprasert, Raweewan Srisawat

**Affiliations:** 1 Department of Medical Entomology, Faculty of Tropical Medicine, Mahidol University, Ratchawithi Road, Ratchathewi, Bangkok 10400, Thailand; pmednty@gmail.com (N.S.); monthatip1020@gmail.com (M.S.); theerawit.pha@mahidol.ac.th (T.P.); patchara.sri@mahidol.ac.th (P.S.); jiraporn.rua@mahidol.ac.th (J.R.); 2 Department of Tropical Hygiene, Faculty of Tropical Medicine, Mahidol University, Ratchawithi Road, Ratchathewi, Bangkok 10400, Thailand; chawarat.rot@mahidol.edu; 3 Mahidol-Oxford Tropical Medicine Research Unit, Faculty of Tropical Medicine, Mahidol University, Ratchawithi Road, Ratchathewi, Bangkok 10400, Thailand

**Keywords:** essential oil, repellent, protection, *Aedes aegypti*, *Anopheles dirus*, *Culex quinquefasciatus*, combination

## Abstract

**Simple Summary:**

The repellent efficacy of ten essential oils was measured against all three-mosquito species using the arm-in-cage approach to determine the duration of protection. All essential oils showed high complete-protection time against *Culex quinquefasciatus,* ranging from 120 to 360 min. Petitgrain oil showed the highest complete-protection time from bites of *Aedes aegypti* for 270 min. Peppermint oil also exhibited complete-protection time from bites of *Anopheles dirus* for 180 min. When two highly effective essential oils were combined to potentially prolong mosquito bite protection, the binary combinations of petitgrain with other essential oils (basil and coriander) exhibited antagonistic effects and provided complete protection against *A. aegypti* bite for 150 min. On the other hand, the sage and patchouli oil combination showed more repellent activity from *A. dirus* bite for 270 min than an individual oil. The binary combination efficiently increased or decreased protection time.

**Abstract:**

Mosquito repellents reduce human-vector contact of vector-borne diseases. We compared the repellent activity of 10 undiluted essential oils (anise, basil, bergamot, coriander, patchouli, peppermint, petitgrain, rosemary, sage and vetiver) against *A. aegypti,* *A. dirus* and *C. quinquefasciatus* using the arm-in-cage method. Petitgrain oil was the most effective against *A. aegypti (*270 min). Peppermint oil was the most effective against *A. dirus* (180 min). Interestingly, all single oils had attributes of repellency against *C. quinquefasciatus* (ranged, 120–360 min). Moreover, we integrated their binary combinations of highly effective essential oils against *A. aegypti* and *A. dirus* to potentially increase the protection time. A 1:1 combination of petitgrain/basil, petitgrain/coriander, basil/coriander and basil/sage reduced the median complete-protection time of 150 min for *A. aegypti*; a combination of sage and patchouli oils prolonged the median complete-protection time of 270 min for *A. dirus*. Combining essential oils effect protection time from these two mosquito species.

## 1. Introduction

Dengue and malaria are mosquito-borne diseases that are widespread in tropical and sub-tropical regions. The primary vectors of dengue, filariasis and malaria are *Aedes*, *Culex* [1,2] and *Anopheles* mosquitoes, respectively. In Thailand, lymphatic filariasis was studied in 23,477 targeted immigrants from 2002 to 2017; the findings revealed that 0.7% of them became seropositive on average [3]. In 2021, there were an estimated 3684 dengue cases [4] and 2950 reported cases of malaria [5].

Personal protection is the simplest method of preventing mosquito bites and reducing mosquito-borne diseases and includes window and door screens, long-sleeved shirts, long trousers, mosquito bed nets and insect repellents [6]. *N*,*N*-diethyl-m-toluamide (DEET) is a synthetic chemical insect repellent that is effective against mosquitoes but can penetrate the skin and cause adverse health effects in humans [7,8,9]. Therefore, there is a need for mosquito repellents that are biodegradable and non-toxic [10]. Multiple studies have investigated natural (plant-based) insect repellents as alternatives to DEET [11]. Essential oils are highly concentrated substances extracted from plants by distillation, and some have been reported to repel mosquitoes of the *Aedes*, *Anopheles*, or *Culex* genera. These include *Ocimum basilicum* (basil), *Citrus bergamia* (bergamot), and *Coriandrum sativum* (coriander) [12,13,14].

Efforts to improve the efficacy of repellents have involved the addition of fixatives such as paraffin, vanillin and coconut oil. Another option would be to combine essential oils for an additive or synergistic effect [11]. In Thailand, citronella oil is the most common active ingredient in plant-based mosquito repellent products such as sprays, lotions and candles. However, the odor of this oil may be too strong for some individuals. In the present study, we assessed the repellent activity of 10 essential oils (anise, basil, bergamot, coriander, patchouli, peppermint, petitgrain, rosemary, sage and vetiver) against *A. aegypti*, *A. dirus* and *C. quinquefasciatus*, as well as their binary combinations against *A. aegypti* and *A. dirus* using the arm-in-cage method under laboratory conditions to identify effective candidates for plant-based mosquito repellents.

## 2. Materials and Methods

### 2.1. Preparation of Essential Oils

The 10 essential oils were selected from the literature on their mosquito-repellent activity, edibility and non-toxicity to humans (Table 1). The 100% pure essential oils were obtained from a reliable supplier, Hong Huat Co., Ltd. (Bangkok, Thailand), and were accompanied by a Certificate of Analysis (COA). Citronella oil was used as a positive control because of its well-known odor and popularity as an herbal insect repellent, particularly against mosquitoes, fleas and biting flies [15].

The essential oils that had (protection time ≥ 120 min and met the FDA Thailand requirement against each mosquito species were selected to be analyzed further in binary combinations (1:1 *v*/*v*). Each combination was specific for each mosquito species listed in Table 2.

### 2.2. Mosquitoes

The free-mating *A. aegypti* (Bora Bora strain) and *C. quinquefasciatus* (Nonthaburi strain), and the forced-mating *A. dirus* (Kao Mai Kaew strain) laboratory-reared females were maintained at the Insecticide Research Unit at the Department of Medical Entomology, Faculty of Tropical Medicine, Mahidol University. Eggs were hatched in plastic trays. After hatching, 200 larvae were reared in plastic trays in 1500 mL of dechlorinated water and fed daily on fish food powder (Optimum, Perfect Companion Group Co., Ltd., Bangkok, Thailand). Pupae were transferred to plastic cups and placed inside a cage (20 × 20 × 30 cm^3^) for emergence. Adult mosquitoes were fed 5% sugar solution on cotton wool, which was changed once a week. All larvae and adult mosquitoes were colonized and maintained under controlled insectary conditions (25 ± 2 °C, 70 ± 10% relative humidity, and 14/10 h light/dark cycle). Both sexes of mosquitos were maintained in the cage to allow for mating [16]. Only *A. aegypti* and *C. quinquefasciatus* are free-mating in the cage, while *A. dirus* requires forced mating. The non-blood-fed female mosquitoes, 5–7 days old *A. aegypti* and *A. dirus*, and 10–12 days old *C. quinquefasciatus* were isolated and sugar fasted for 12 h before testing but had access to limited water. Female mating or unmating had no effect on blood feeding [17].

The age of female mosquitoes was considered in this study in accordance with WHO recommendations [16] that they should be host-seeking in a uniform cage, which was usually implemented, preferably 5–7 days post-emergence. However, 10–12-day-old *C. quinquefasciatus* were more active in seeking hosts than 5–7-day-old, resulting in more than 10 bites on the control arm within 3 min.

### 2.3. Human Volunteers

Five male and female volunteers were 20–65 years old, healthy, with no history of an allergic reaction to mosquito bites. We excluded women who were pregnant and individuals who were sensitive to essential oils or taking daily medication. Volunteers did not use fragrance and repellent products for 12 h before the experiment and during testing. Volunteers were not tobacco users or at least had refrained from tobacco use for 12 h before and during testing. Volunteers were interviewed and instructed in the methodology. Each volunteer signed an informed consent form.

### 2.4. Repellent Activity Test

Undiluted essential oils were evaluated for repellency against female *A. aegypti, A. dirus* and *C. quinquefasciatus* using the arm-in-cage technique modified from the standard World Health Organization method [16] and Junkum et al. [18]. Each essential oil was tested on adult healthy volunteers at random. One volunteer was tested once per essential oil, once per mosquito species, and once per day. The test area of the human volunteer (inner part of the forearm between wrist and elbow) was washed with unscented soap and 70% ethanol and covered with a rubber sleeve with a 3 × 10 cm window. The hand was protected with a glove. Approximately 100 µL of undiluted essential oil was dropped on gauze and allowed to dry for 1 min, and the gauze was rubbed on a test area of skin. The other forearm served as a control and was exposed to mosquitoes without the application of essential oil. Fifty fasted aged 5–7 days *A. aegypti* and *A. dirus* females, and 250 fasted aged 10–12 days *C. quinquefasciatus* were selected at random and placed in a standard cage (30 × 30 × 30 cm^3^) and rested for 1–2 h before the experiment. Tests were conducted by exposing the control and tested forearms in the test cage containing mosquitoes. The control arm was exposed before the treatment arm. If at least 10 mosquitoes landed on the control arm within 3 min, the repellency test was continued. After 30 min post application, the tested arm was exposed for 3 min, then withdrawn for 30 min, and then exposed again. The experiment was stopped when the second mosquitoes landed on the test area and recorded as the complete-protection time for that subject [18]. The protection time was measured between the application of essential oil and the second landed. The Thai Food and Drug Administration (Thai FDA) determined that protection time for *A. aegypti* should be at least 120 min [19], which is the regular criterion used to consider repellent efficacy. The exposure period was determined by the biting behavior of the mosquitos; *A. aegypti* and *A. dirus* were exposed from 8:00 to 15:00 h, and *C. quinquefasciatus* from 15:00 to 22:00 h.

*C. quinquefasciatus* prefers to bite at night and is less anthropophilic [20]. Then, 250 mosquitoes were the appropriate number to conduct the experiment resulting in more than 10 bites landing on the control arm within 3 min, and this number was not higher than the WHO guidelines. Furthermore, because there was no comparison among species, the different numbers of each species had no effect on the results of each essential oil within species.

### 2.5. Data Analysis

The complete protection time of each volunteer was recorded. These data were non-normally distributed and were small samples; therefore, the differences in the protection time and the type of essential oil were analyzed by using the non-parametric Kruskal–Wallis test. If the ANOVA was significant, Dunn’s multiple comparisons were performed with the Benjamini–Hochberg correction at a critical level of 0.05 (PASW Statistics for Windows, version 18.0; IBM SPSS, Armonk, NY, USA).

## 3. Results

### 3.1. Repellent Activity of Individual Essential Oils against A. aegypti

**Figure 1:**
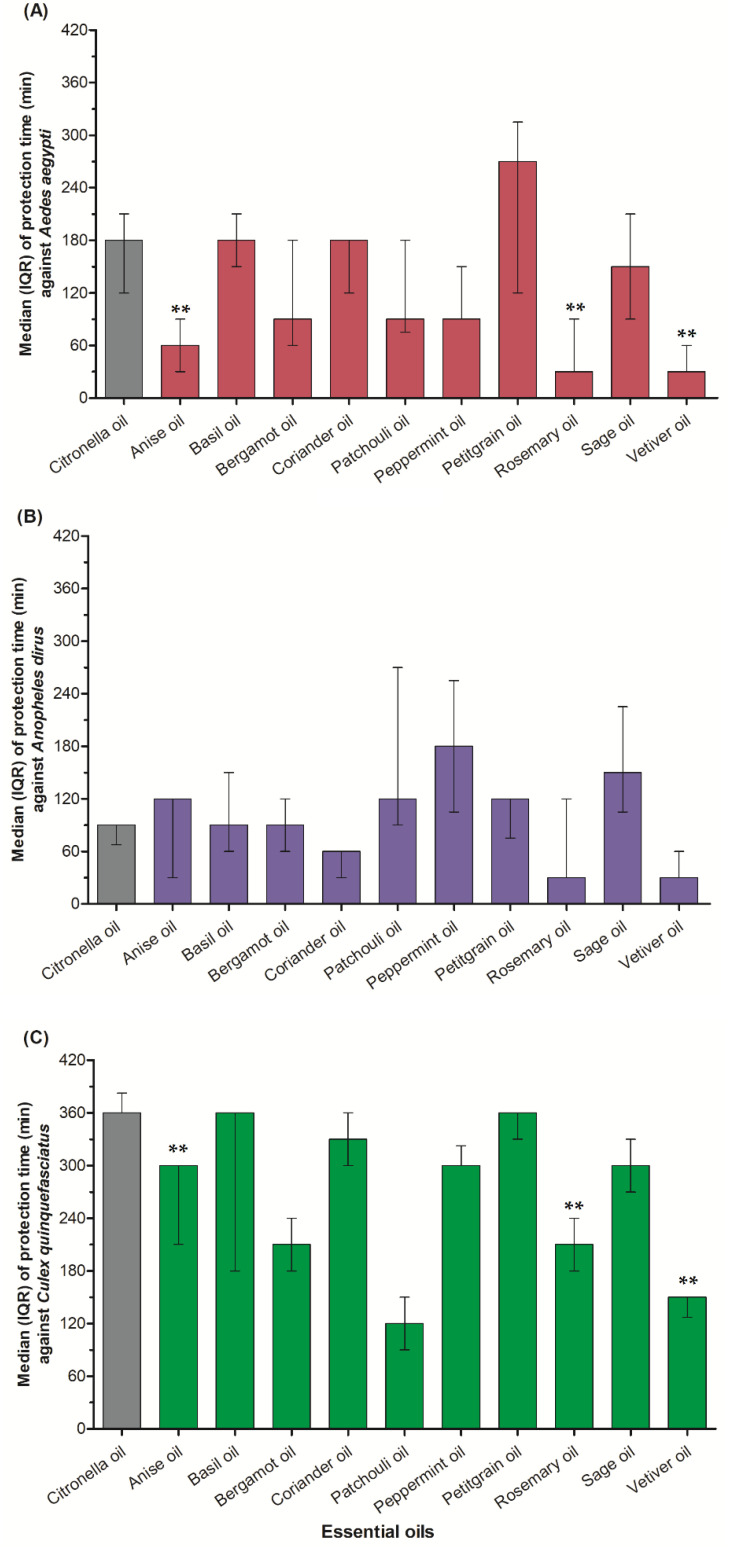
Median with interquartile range (IQR) protection time of 10 undiluted essential oils and citronella (control) against (**A**) *Aedes aegypti*, (**B**) *Anopheles dirus* and (**C**) *Culex quinquefasciatus*. ** Significantly different from control (*p* < 0.05).

We assessed the repellent activity of 10 tested essential oils against *A. aegypti* by the arm-in-cage method (Figure 1A). Oils that provided protection for 120 min were considered highly effective; these were the essential oils of petitgrain (270 min), basil (180 min), coriander (180 min) and sage (150 min). The other essential oils had much lower median complete-protection times of 90 min (bergamot, patchouli and peppermint), 60 min (anise) and 30 min (rosemary and vetiver). The protection time was significantly different (Chi-square = 22.60, *p* = 0.012, df = 10) among the individual essential oils. The results of pairwise comparisons of individual essential oils (Table S1) against *A. aegypti* were provided in Table S2A in the supplementary document.

### 3.2. Repellent Activity of Individual Essential Oils against A. dirus

The median protection time of 10 undiluted tested essential oils against *A. dirus* under laboratory conditions (Figure 1B). When compared with citronella oil (90 min) as a control, only five essential oils (peppermint, sage, anise, patchouli and petitgrain) were highly effective over 120 min against the mosquito, with median complete-protection times of 180, 150, 120, 120 and 120 min, respectively. Basil, bergamot, coriander, rosemary and vetiver had low median protection times of 90, 90, 60, 30 and 30 min, respectively. The protection time was significantly different (Chi-square = 20.08, *p* = 0.029, df = 10) among the individual essentials. The complete results of pairwise comparisons of individual essential oils against *A. dirus* were provided in Table S2B in the supplementary document.

### 3.3. Repellent Activity of Individual Essential Oils against C. quinquefasciatus

Followed by FDA Thailand requirements, the repellent time against mosquitoes should be more than 120 min; all 10 tested essential oils showed high levels of protection (Figure 1C), with median complete-protection times of 360 min (petitgrain, citronella and basil), 330 min (coriander), 300 min (peppermint, sage and anise), 210 min (bergamot and rosemary), 150 min (vetiver) and 120 min (patchouli). The protection time was significantly different (Chi-square = 29.46, *p* = 0.001, df = 10) among the individual essential oils. The results of pairwise comparisons of individual essential oils against *C. quinquefasciatus* were provided in Table S2C in the supplementary document.

### 3.4. Repellent Activity of Essential Oil Combinations against A. aegypti

**Figure 2:**
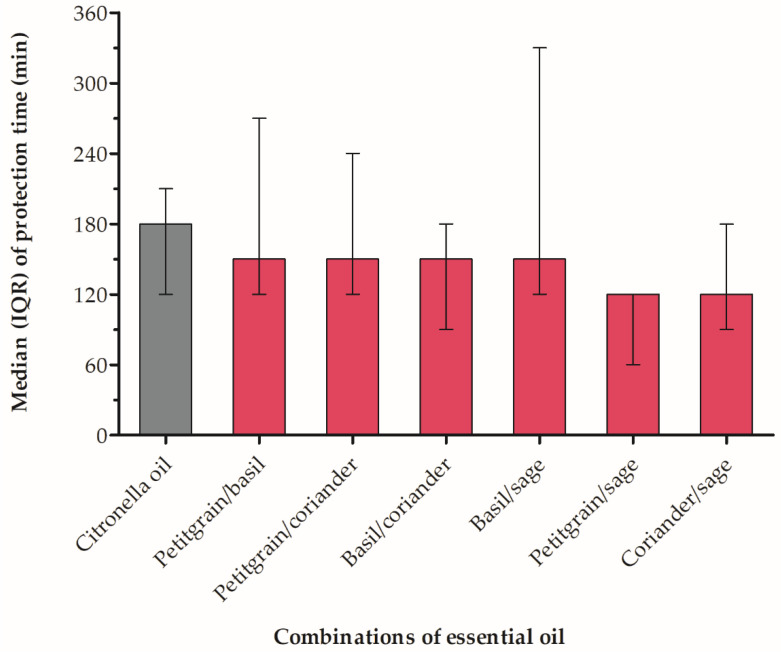
Median with interquartile range (IQR) protection times of essential oil combinations and citronella oil (control) against *Aedes aegypti*.

We mixed the four essential oils (petitgrain, basil, coriander and sage) in two-oil combinations against *A. aegypti* at a 1:1 (*v*/*v*) ratio to evaluate whether they exerted additive or synergistic effects (Table 2). All combinations provided more than 120 min of protection (Figure 2). A median complete-protection time of 150 min was observed for petitgrain/basil, petitgrain/coriander, basil/coriander and basil/sage combinations. A median complete-protection time of 120 min was observed for petitgrain/sage and coriander/sage combinations. All six binary combinations evaluated against *A. aegypti* exhibited an antagonistic effect, showing that combined had a lower repellent efficacy than individual oil applications and citronella oil (180 min). The combinations were not significantly different in their efficacy against *A. aegypti* (Chi-square = 4.47, *p* = 0.483, df = 5). Since the analysis of variance was not significant, the post-hoc comparisons were not performed.

### 3.5. Repellent Activity of Essential Oil Combinations against A. dirus

**Figure 3:**
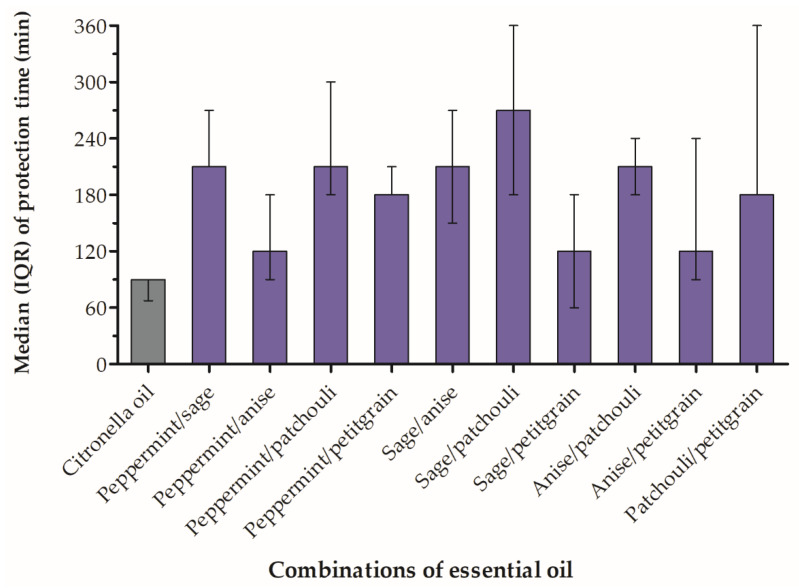
Median with interquartile range (IQR) protection times of essential oil combinations and citronella (control) against *Anopheles dirus*.

The five essential oils (peppermint oil, sage oil, anise oil, patchouli oil and petitgrain oil) were blended into two-oil against *A. dirus* at a 1:1 (*v*/*v*) ratio to evaluate whether they exerted additive or synergistic effects (Table 2). All combinations provided more than 120 min of protection (Figure 3). Combining sage and patchouli oil resulted in the highest median complete-protection time (270 min). A median complete-protection time of 210 min was observed for peppermint/sage, peppermint/patchouli, sage/anise and anise/patchouli combinations. A median complete-protection time of 180 min was observed for peppermint/petitgrain and patchouli/petitgrain combinations. A median complete-protection time of 120 min was observed for peppermint/anise, sage/petitgrain and anise/petitgrain combinations. The combinations were not significantly different in their efficacy against *A. dirus* (Chi-square = 12.79, *p* = 0.172, df = 9). However, the combined essential oil prolonged repellency against *A. dirus* over that of individual oils. Because the non-parametric Kruskal–Wallis test was not significant, the pairwise comparisons were not calculated.

## 4. Discussion

Our research clearly revealed that some essential oils studied were effective against *A. aegypti, A. dirus* and *C. quinquefasciatus* to some degree in the human arm-in-cage assay. Few essential oils examined have higher repellent efficacy than citronella, the well-known natural mosquito repellent in Thailand [21]. The FDA Thailand minimum requirement for mosquito-bite protection was 120 min for *A. aegypti*. Conversely, the FDA Thailand does not prescribe for other mosquito species, and when considering the repellent effect of citronella oil on *A. dirus*, the protection time of 90 min was revealed, which was consistent with previous studies [12,22,23]. Against *C. quinquefasciatus,* all tested essential oils showed repellent potential with protection time higher than 120 min. Petitgrain and basil oil exhibited the highest repellency, with the protection time of 360 min equaled to citronella. This was in concordance with earlier studies [24,25]. We considered that the essential oils alone were sufficient enough to repel *C. quinquefasciatus*. Combining them to improve repelling duration is unnecessary in this study. However, this is a significant benefit that we will study further by integrating it with other product and packaging technologies, featuring a prototype product that is reliable and comfortable for users. Above all, we directly targeted further development of plant-based repellents for anti-mosquito vectors in dangerous vector-borne diseases, urban (*A. aegypti*), forest and jungle areas (*A. dirus*).

Petitgrain oil was the most effective against *A. aegypti*, in line with previous reports [26,27,28]. Essential oils from basil, coriander and sage were also effective in repelling *A. aegypti* and are known to contain insect-repelling compounds such as α-terpineol, citral, eugenol, linalool, menthol and estragole [29,30,31]. Diluted basil oil was previously shown to repel *A*. *aegypti*, *A*. *dirus,* and *C. quinquefasciatus* [23,32]. The major constituent of coriander oil is linalool, and the oil has been shown to be effective for 60 min against *A. albopictus* [14]. Sage oil showed biting-deterrent activity against *A*. *aegypti* and *A. quadrimaculatus* [33]; its main constituents are viridiflorol, α-pinene, α and β-thujone, camphor and 1,8-cineole [33,34,35].

The 100% peppermint oil exhibited the highest efficacy against *A. dirus* (180 min), longer than the 64 min recorded for 10% peppermint oil in soybean oil [23]. A previous study reported 100% protection by peppermint oil against *A. aegypti* for 150 min [10]; the major constituents of peppermint oil are limonene, α-pinene, myrcene and linalool [36]. We also observed good efficacy (>120 min) against *A. dirus* by essential oils of sage, anise, patchouli and petitgrain. This finding is consistent with previous reports for patchouli against *A. dirus* [22] and sage against another malaria vector, *A. stephensi* [34]. Patchouli oil contains patchoulol, α-bulnesene, α-guaiene and pogostone [37,38], as well as limonene, linalool and β-myrcene [39]. Petitgrain oil has been shown as highly effective against *A. dirus* in combination with vanillin [28]. The major constituents of anise oil are trans-anetole, estragole, γ-hymachalen and para-anisaldehyde [40], and it has been shown to repel *A. stephensi*, *A. aegypti*, and *C. quinquefasciatus* [41].

The repellent activity of essential oils depends on their chemical constituents [28]. Camphor, cineol, linalool and α-pinene have been identified as insect repellents [42,43,44,45,46,47]. Limonene and linalool act as contact poisons or nerve toxins [48]. Essential oils are composed mainly of monoterpenes, sesquiterpenes, alcohols and phenols that can be neurotoxic to mosquitoes [49]. However, the use of essential oils as insect repellents is limited by their volatility and oxidation. Stabilizing the oils may involve additives such as vanillin, coconut oil and olive oil [28,32] to extend protection times and substitutes for synthetic repellents [15,27,50]. *Lantana camara* flower extract in coconut oil was shown to protect against *A. albopictus* and *A. aegypti* without side effects in human volunteers [51], while a combination of 5% eucalyptus oil and 5% basil oil in soybean oil protected against both *A*. *aegypti* and *A*. *dirus* [23]. According to these studies, combining essential oils resulted in a longer duration of mosquito protection time than applying them alone. However, in this report, the repellency of *A. aegypti* was shown to be lower in all six binary combinations than in single oils, suggesting that some components present in each essential oil have antagonistic effects or interact antagonistically with each other [52].

We tested binary mixtures of the most effective essential oils for additive or synergistic effects. Petitgrain/basil, petitgrain/coriander, basil/coriander and basil/sage combinations were shown to be efficient against *A. aegypti* for 150 min, and the combination of sage and patchouli was most effective against *A. dirus* (270 min). Although no synergistic interaction was found when considering the repellency of oil combinations in repelling *A. aegypti*, antagonism in the repellent activity of plant essential oils or their main components is not unusual. In particular, as compared to those single oils, the repellent efficacy of the binary blends of sandalwood oil with marjoram, cypress, lemongrass and clary sage oil revealed an antagonistic effect to repel spider mite, *Tetranychus urticae* [53]. Monoterpene compound binary combinations exhibited antagonistic interactions with *Spodoptera littoralis* larvae in 150 of the 435 combinations [54] and *C. quinquefasciatus* larvae in 74 of the 435 combinations [55]. Interestingly, when the most active oils in repellent activity against *n. dirus* were combined, they improved the median complete-protection time and had a synergistic activity. A similar trend in synergistic effect was found in an increase in the mean protection time against female *Stomoxys calcitrans* generated by binary combinations of patchouli and tamanu oil (138 min) compared to patchouli oil (41.4 min) or tamanu oil (33.3 min) alone [56]. Additionally, a 1:1 mixture of eucalyptus and clove essential oils was shown to exhibit better protection than individual oils against *Anopheles* mosquito [57]. Therefore, when two single oils were mixed, the type, quantity or ratio of active ingredients changed. This may increase or decrease the effective protection time against mosquito bites. To fully comprehend the synergistic or antagonistic response when plant essential oils are applied, a comprehensive study of all possible factors is necessary.

Finally, the different species of mosquitoes have different repellent responses to essential oils [58]. Essential oil is differently stimulated by olfactory receptors of mosquitoes. Over millions of years of evolution, the mosquito chemosensory receptors have diversified to the point where one species may be able to detect a chemical that encourages repulsion while others cannot. Adult *Aedes* and *Culex* mosquitoes, for instance, appear to express odorant receptors that respond to DEET but are not expressed in *Anopheles* [59].

In this study, *C. quinquefasciatus* showed an easier repel response with essential oil than *A. aegypti* and *A. dirus*, which is similar to other reports [59,60]. *C. quinquefasciatus* was sensitive to all 41 essential oils tested, but *A. aegypti* was tolerable to several oils [59] due to its aggressive biting behavior [61]. This finding can be explained by the fact that *C. quinquefasciatus* feed on animals and can seek blood meal elsewhere while being rejected by human hosts [62], but *A. aegypti* and *A. dirus* are extremely anthropophilic, meaning they more frequently attack humans [59,62].

Essential oil-based repellents are considered safer and more accessible than synthetic chemical repellents and may have a lower probability of resistance development in mosquitoes. Plant-based mosquito repellents can be applied to skin or clothing without affecting non-target organisms or the environment [63]. In the present study, all tested essential oils exhibited high repellency against *C. quinquefasciatus,* petitgrain and basil oils being the most potential. The combinations of petitgrain/basil, petitgrain/coriander, basil/coriander and basil/sage were responsible for repelling *A. aegypti* bite, although the repellent efficiency was lower than that of the individual oils, and sage/patchouli combinations yielded effective and long-lasting protection against *A. dirus*. These combinations may be candidates for the formulation of safe and effective mosquito repellents. The synergistic repellent effect of the essential oils used in this study may contribute to the development of more effective alternatives to synthetic repellents for human mosquito protection.

## 5. Conclusions

Petitgrain, peppermint and basil oil were highly effective against female *A. aegypti* and *C. quinquefasciatus*. Combining petitgrain/basil, petitgrain/coriander, basil/coriander and basil/sage showed efficacy against *A. aegypti*, as did a combination of sage and patchouli oils against *A. dirus.* Field trials should include a study of operational feasibility and dermal toxicity before these essential oil combinations can be developed as alternative and safe insect repellents for individual use to reduce and control vector-borne diseases.

## Figures and Tables

**Table 1:** Essential oils used in the study.

Scientific Name	Common Name	Family	CAS No.
*Cymbopogon nardus*	citronella	Poaceae	91771-61-8
*Pimpinella anisum*	anise	Apiaceae	84775-42-8
*Ocimum basilicum*	basil	Lamiaceae	8015-73-4
*Citrus bergamia*	bergamot	Rutaceae	8007-75-8
*Coriandrum sativum*	coriander	Apiaceae	8008-52-4
*Pogostemon cablin*	patchouli	Lamiaceae	8014-09-03
*Mentha piperita*	peppermint	Lamiaceae	8030-01-1
*Citrus aurantium*	petitgrain	Rutaceae	8014-17-3
*Rosmarinus officinalis*	rosemary	Lamiaceae	8000-25-7
*Salvia officinalis*	sage	Lamiaceae	8022-56-8
*Vetiveria zizanioides*	vetiver	Poaceae	84238-29-9

**Table 2:** Essential oil combinations used in the study.

*Aedes aegypti* Protection	*Anopheles dirus* Protection
petitgrain and basil	peppermint and sage
petitgrain and coriander	peppermint and anise
petitgrain and sage	peppermint and patchouli
basil and coriander	peppermint and petitgrain
basil and sage	sage and anise
coriander and sage	sage and patchouli
	sage and petitgrain
	anise and patchouli
	anise and petitgrain
	patchouli and petitgrain

## Data Availability

The data presented in this study are available in article and supplementary material.

## Supplementary Materials

The following supporting information can be downloaded at: https://www.mdpi.com/article/10.3390/insects13070658/s1, Tabel S1: Labels of essential oil used in study; Table S2A: Pairwise comparisons of undiluted essential oils and citronella (control) against *Aedes aegypti*; Table S2B: Pairwise comparisons of undiluted essential oils and citronella (control) against *Anopheles dirus*; Table S2C: Pairwise comparisons of undiluted essential oils and citronella (control) against *Culex quinquefasciatus*.
