# Correction: Sutthanont et al. Effectiveness of Herbal Essential Oils as Single and Combined Repellents Against *Aedes aegypti*, *Anopheles dirus* and *Culex quinquefasciatus* (Diptera: Culicidae). *Insects* 2022, *13*, 658

**DOI:** 10.3390/insects17060565

**Published:** 2026-05-29

**Authors:** Nataya Sutthanont, Monthatip Sudsawang, Theerawit Phanpoowong, Patchara Sriwichai, Jiraporn Ruangsittichai, Chawarat Rotejanaprasert, Raweewan Srisawat

**Affiliations:** 1 Department of Medical Entomology, Faculty of Tropical Medicine, Mahidol University, Ratchawithi Road, Ratchathewi, Bangkok 10400, Thailand; pmednty@gmail.com (N.S.); monthatip1020@gmail.com (M.S.); theerawit.pha@mahidol.ac.th (T.P.); patchara.sri@mahidol.ac.th (P.S.); jiraporn.rua@mahidol.ac.th (J.R.); 2 Department of Tropical Hygiene, Faculty of Tropical Medicine, Mahidol University, Ratchawithi Road, Ratchathewi, Bangkok 10400, Thailand; chawarat.rot@mahidol.edu; 3 Mahidol-Oxford Tropical Medicine Research Unit, Faculty of Tropical Medicine, Mahidol University, Ratchawithi Road, Ratchathewi, Bangkok 10400, Thailand

There was an error in the original publication [1]. The ethics approval information in the Institutional Review Board Statement was not accurately presented. The human ethics approval was reported using the EC submission number rather than the Certificate of Ethical Approval number, and the animal ethics approval was reported using the protocol number rather than the approved number.

A correction has been made to the Institutional Review Board Statement:

“The study was conducted according to the guidelines of the Ethics Committee of the Faculty of Tropical Medicine, Mahidol University, under Certificate of Ethical Approval No. MUTM 2018-064-03 for human subjects (arm-in-cage method), and Approved No. FTM-ACUC 029/2018 for animal use by the Institutional Animal Care and Use Committee of the Faculty of Tropical Medicine, Mahidol University (FTM-ACUC), Bangkok, Thailand.”

The authors state that the scientific conclusions are unaffected. This correction was approved by the Academic Editor. The original publication has also been updated.
