# Supplementary material for: Effectiveness of Herbal Essential Oils as Single and Combined Repellents against Aedes aegypti, Anopheles dirus and Culex quinquefasciatus (Diptera: Culicidae)
Source: Insects. 2022 Jul 21;13(7):658. doi: 10.3390/insects13070658 (PMC9322308; doi:10.3390/insects13070658)
Supplement: Supplementary file 1 [file insects-13-00658-s001.zip › insects-1783805-supplementary.pdf]

Supplementary information: Effectiveness of herbal essential oils as single and combined repellents against *Aedes aegypti, Anopheles dirus* and *Culex quinquefascuatus* (Diptera: Culicidae)

Essential oil pairwise results were evaluated with Dunn's test for multiple comparisons, corrected with the Benjamini–Hochberg correction. Significant differences between essential oils were indicated by * at FWER = 0.1 and ** at FWER = 0.05. The labels of each oil were indicated in Table S1. Multiple comparisons of 10 undiluted essential oils and citronella (control) were shown in Table S2A for *Aedes aegypti,* Table S2B for *Anopheles dirus* and Table S2C *Culex quinquefasciatus*. The top row in each cell of pairwise comparison results represents the test statistics whereas the bottom represents the *p*-values.

Table S1. Labels of essential oil used in this study.

Oil		Type
1	Citronella oil (control)	Single
2	Anise oil	Single
3	Basil oil	Single
4	Bergamot oil	Single
5	Coriander oil	Single
6	Patchouli oil	Single
7	Peppermint oil	Single
8	Petitgrain oil	Single
9	Rosemary oil	Single
10	Sage oil	Single
11	Vetiver oil	Single

Table S2A Pairwise comparisons of undiluted essential oils and citronella (control) against *Aedes aegypti*.

	1	10	11	2	3	4	5	6	7	8
10	-0.03628<br>0.4855									
11	2.194869<br>0.0775*	2.231148<br>0.0784								
2	1.741384<br>0.132	1.777663<br>0.1383	-0.4534<br>0.4064							
3	-0.58046<br>0.3677	-0.54418<br>0.375	-2.7753<br>0.0506*	-2.32185<br>0.0795*						
4	0.770658<br>0.3277	0.811219<br>0.3278	-1.6838<br>0.141	-1.17627<br>0.2352	1.419634<br>0.1862					
5	-0.30837<br>0.453	-0.27209<br>0.4409	-2.5032<br>0.0564*	-2.04975<br>0.101	0.272091<br>0.4501	-1.11543<br>0.2348				
6	0.807163<br>0.3205	0.847724<br>0.3208	-1.6467<br>0.1442	-1.13976<br>0.2332	1.456138<br>0.1817	0.042152<br>0.5014	1.151931<br>0.2365			
7	0.653019<br>0.3446	0.689297<br>0.3373	-1.5415<br>0.1693	-1.08837<br>0.2376	1.23348<br>0.2214	-0.04056<br>0.4928	0.961389<br>0.2803	-0.07707<br>0.4964		
8	-0.73821<br>0.3332	-0.69765<br>0.3423	-3.1925<br>0.0388**	-2.68514<br>0.0498**	-0.08923<br>0.5009	-1.74229<br>0.14	-0.39344<br>0.4241	-1.78444<br>0.146	-1.46831<br>0.186	
9	1.922778<br>0.1153	1.959057<br>0.1148	-0.2709<br>0.4596	0.181394<br>0.4708	2.503239<br>0.0677*	1.379073<br>0.1924	2.231148<br>0.0882*	1.342568<br>0.1974	1.269759<br>0.2159	2.887941<br>0.0533*

Table S2B Pairwise comparisons of undiluted essential oils and citronella (control) against *Anopheles dirus.*

	1	10	11	2	3	4	5	6	7	8
10	-1.77377<br>0.1744									
11	1.115381<br>0.28	2.795807<br>0.0712*								
2	-0.32965<br>0.4434	1.28456<br>0.2736	-1.3517<br>0.2554							
3	-0.51028<br>0.3993	1.095654<br>0.2591	-1.5206<br>0.2353	-0.16896<br>0.4492						
4	-0.2574<br>0.4472	1.360122<br>0.2655	-1.2841<br>0.2607	0.067585<br>0.4731	0.236547<br>0.4384					
5	0.862501<br>0.3142	2.531339<br>0.0625*	-0.2365<br>0.4472	1.115152<br>0.2697	1.284115<br>0.2489	1.047567<br>0.2615				
6	-1.50231<br>0.2286	0.287931<br>0.4431	-2.5464<br>0.0748*	-1.0352<br>0.2583	-0.8463<br>0.3122	-1.11077<br>0.2619	-2.2819<br>0.0884*			
7	-1.9712<br>0.1488	-0.2094<br>0.4411	-2.9771<br>0.08*	-1.46591<br>0.2308	-1.277<br>0.231	-1.54147<br>0.242	-2.7126<br>0.0612*	-0.49734<br>0.3958		
8	-0.76195<br>0.3228	1.073198<br>0.2596	-1.8663<br>0.155	-0.35514<br>0.4415	-0.16624<br>0.442	-0.43071<br>0.4167	-1.6019<br>0.2309	0.785267<br>0.3213	1.282602<br>0.2387	
9	0.519307<br>0.4048	2.172417<br>0.1025	-0.5575<br>0.3968	0.794124<br>0.3263	0.963086<br>0.2796	0.726539<br>0.3297	-0.3213<br>0.4378	1.923062<br>0.1498	2.353767<br>0.0852*	1.243<br>0.2353

Table S2C Pairwise comparisons of undiluted essential oils and citronella (control) against *Culex quinquefasciatus.*

	1	10	11	2	3	4	5	6	7	8
10	1.483844<br>0.1723									
11	3.788799<br>0.0042**	2.023901<br>0.0844								
2	1.882333<br>0.0967*	0.372751<br>0.3981	-1.62541<br>0.1363							
3	1.085356<br>0.2634	-0.37275<br>0.4064	-2.42239<br>0.0424**	-0.7455<br>0.3135						
4	2.61639<br>0.0305**	1.059399<br>0.2567	-0.89136<br>0.2847	0.686647<br>0.3223	1.432151<br>0.1673					
5	0.833679<br>0.3006	-0.60817<br>0.3473	-2.67407<br>0.0343**	-0.98093<br>0.2642	-0.23542<br>0.4389	-1.66757<br>0.1312				
6	3.727963<br>0.0027**	2.09918<br>0.0757*	0.220217<br>0.4367	1.726429<br>0.1287	2.471932<br>0.0411**	1.039781<br>0.2487	2.707354<br>0.0373**			
7	1.461151<br>0.1721	-0.13108<br>0.4561	-2.32765<br>0.0457**	-0.52957<br>0.3645	0.267406<br>0.434	-1.26363<br>0.2102	0.519083<br>0.3609	-2.3752<br>0.0438**		
8	0.602975<br>0.3416	-0.82398<br>0.2967	-2.90477<br>0.0253**	-1.19673<br>0.2273	-0.45123<br>0.3814	-1.88338<br>0.1025	-0.2158<br>0.4302	-2.9231<br>0.0318**	-0.74979<br>0.3197	
9	2.61639<br>0.0349**	1.059399<br>0.2653	-0.89136<br>0.2929	0.686647<br>0.3302	1.432151<br>0.1743	0<br>0.5	1.667573<br>0.1381	-1.0397<br>0.2565	1.263627<br>0.2183	1.883377<br>0.1094
